# Using Machine Learning to Predict Cognitive Impairment Among Middle-Aged and Older Chinese: A Longitudinal Study

**DOI:** 10.3389/ijph.2023.1605322

**Published:** 2023-01-19

**Authors:** Haihong Liu, Xiaolei Zhang, Haining Liu, Sheau Tsuey Chong

**Affiliations:** ^1^ Centre for Research in Psychology and Human Well-being, Faculty of Social Sciences and Humanities, Universiti Kebangsaan Malaysia, Bangi, Malaysia; ^2^ Department of Psychology, Chengde Medical University, Chengde, China; ^3^ Department of Biomedical Engineering, Chengde Medical University, Chengde, China; ^4^ Faculty of Engineering, Universiti Putra Malaysia, Serdang, Malaysia; ^5^ Hebei Key Laboratory of Nerve Injury and Repair, Chengde Medical University, Chengde, China; ^6^ Hebei International Research Center of Medical Engineering, Chengde Medical University, Chengde, China; ^7^ Counselling Psychology Programme, Secretariat of Postgraduate Studies, Faculty of Social Sciences and Humanities, Universiti Kebangsaan Malaysia, Bangi, Malaysia

**Keywords:** longitudinal study, machine learning, random forest, middle-aged and older Chinese, cognitive impairment, dementia

## Abstract

**Objective:** To explore the predictive value of machine learning in cognitive impairment, and identify important factors for cognitive impairment.

**Methods:** A total of 2,326 middle-aged and elderly people completed questionnaire, and physical examination evaluation at baseline, Year 2, and Year 4 follow-ups. A random forest machine learning (ML) model was used to predict the cognitive impairment at Year 2 and Year 4 longitudinally. Based on Year 4 cross-sectional data, the same method was applied to establish a prediction model and verify its longitudinal prediction accuracy for cognitive impairment. Meanwhile, the ability of random forest and traditional logistic regression model to longitudinally predict 2-year and 4-year cognitive impairment was compared.

**Results:** Random forest models showed high accuracy for all outcomes at Year 2, Year 4, and cross-sectional Year 4 [AUC = 0.81, 0.79, 0.80] compared with logistic regression [AUC = 0.61, 0.62, 0.70]. Baseline physical examination (e.g., BMI, Blood pressure), biomarkers (e.g., cholesterol), functioning (e.g., functional limitations), demography (e.g., age), and emotional status (e.g., depression) characteristics were identified as the top ten important predictors of cognitive impairment.

**Conclusion:** ML algorithms could enhance the prediction of cognitive impairment among the middle-aged and older Chinese for 4 years and identify essential risk markers.

## Introduction

With the current rapidly aging global population, the burden of dementia in low-income countries is expected to increase dramatically in the coming decades [1]. Currently, 47.47 million people worldwide have been diagnosed with dementia but by 2050, this number is expected to double [2]. To identify the population with the highest risk of dementia, focusing on the early stages of the pathological process is a viable strategy for prevention. Cognitive impairment is characterized by decreased memory, attention and language, and deterioration in other cognitive functions, including mild cognitive impairment and dementia [3]. At present, neuropsychological assessment is an important method for screening and diagnosing cognitive impairment. For example, neuropsychological examinations such as Mini-mental State Examination (MMSE) and Montreal Cognitive Assessment (MoCA) are useful evaluation tests for cognitive function [5, 6]. Considering the shortage of community professionals and time, telephone interview for cognitive status (TICS) with fewer items has demonstrated its value as an effective screening tool for cognitive impairment in the community compared with other methods such as MMSE [4]. This method complements commonly used cognitive function evaluation tools, by identifying potential risk factors for cognitive impairment. Early screening of individual cognitive impairment is crucial to preventing cognitive decline, and progression to dementia through immediate effective treatment and management strategies.

At present, a fundamental strategy to prevent or minimize cognitive decline is through early detection of the risk factors for cognitive impairment, which benefits preventive intervention [5]. Therefore, the prediction of cognitive impairment plays an important role in mitigating and preventing cognitive impairment. However, predicting cognitive impairment is a challenging process. Few empirical studies have compared the predictors of cognitive impairment, and these methods mainly used meta-analysis methods and traditional regression methods. For example, a meta-analysis reported in the Lancet in 2020, revealed that approximately 35% of dementia was contributed by nine factors, including previous education, high blood pressure, middle-aged obesity, and late-life depression [5]. Researchers have used traditional regression statistical methods to identify the common predictors of cognitive impairment outcomes including demographic characteristics and general health information. However, these studies did not point out the importance ranking of influencing factors.

Notably, in these studies, the most commonly used analysis method was regression-based inferential statistics. Yet, the predictors produced by these surveys were insensitive [6]. Several issues associated with conventional statistics limit their robust prediction of complex neurodegenerative processes. Traditional regression-related approaches can only accommodate a restricted number of predictors and cannot process the complex multi-class characteristic variables [7]. In addition, these methods are based on linear assumptions and may not be able to effectively manage more complex patterns including non-linear and higher dimensional [8].

Emerging computing methods using machine learning can optimize the prediction of cognitive impairment, to overcome the shortcomings of traditional methods. Machine learning(ML) has been used for clinical classification and prediction based on extracted high-dimensional features from data [9]. The random forest is a typical ML technique with high predictive performance and robustness as regards to its accuracy and ease of implementation [10, 11]. This method has a high level of predictive ability. It creates multiple decision trees by implementing random sampling in the same data set, combining them and finally predicting the target variable [12, 13]. Importantly, random forests also have excellent predictive ability to discover the correlation between explanatory variables and diseases, while preventing over-fitting when multiple explanatory variables are applied to the model [14, 15]. Compared with other methods, random forest demonstrated the highest accuracy compared with other methods in predicting cognitive impairment [16, 17].

In recent years, machine learning, has been increasingly used in research to predict cognitive impairment. These studies mainly have two characteristics. First, most of these studies use expensive sample data sources, such as MRI, PET and other medical imaging methods [18–20]. Second, predictor variables in some studies that predict cognitive impairment or other diseases, are only self-reported variables [16, 21, 22].

Cognitive impairment is an age-related condition caused by Alzheimer’s disease, vascular dementia, mixed dementia or other related types of dementias with no cure [23, 24]. Therefore, ML technology has high potential value in evaluation of risk factors of cognitive impairment. Conventional regression-based approaches have been used to effectively identify key risk factors, including demographic (e.g., education, venerable age, gender) [25], physical condition (e.g., body mass index (BMI), hearing difficulty) [26, 27], lifestyle activities (e.g., smoking, drinking and instrumental activities of daily living (IADL) [28], poor psychological wellbeing (e.g., depression) [29–31], and chronic diseases (e.g., chronic lower back pain) [32]. However, few studies have examined the effect of these independent risk factors on cognitive impairment among older Chinese using a prospective design.

Given the potentially severe consequences of cognitive impairment, an improvement in the predictions for middle-age and older people is important. To solve this problem and fill gaps in the previous literature, this study aims to examine the predictive power of the ML model for cognitive impairment using China Health and Retirement Longitudinal Survey database. In addition, the study compared the prediction accuracy of random forest in ML with traditional inferential statistical method (logistic regression). First, a large representative elderly sample, which consisted of middle-aged and elderly people, participated in a 3-year survey. Second, random forest and logistic regression were used to longitudinally predict cognitive impairment and identify ten most important predictors, including biological factors and psychological at 2-year and 4-year follow-ups from 44 baseline predictors. These baseline predictors included demographic (e.g., age, education level), health status and functioning (e.g., physical functions, biomarkers), emotional status (e.g., depression), lifestyle and behavior (e.g., smoking, drinking, sleeping habits). Finally, a 4-year follow-up cross-sectional data was applied to construct a model to verify the accuracy of the longitudinal prediction model for previous 2 years.

It was assumed that the random forest model of ML could predict long-term cognitive impairment. In addition, compared with the logistic regression model, the random forest model could improve the prediction of long-term cognitive impairment outcomes. ML model could also screen out the risk factors with the most significant impact on cognitive impairment. Overall, the study results provide insights into the practicability of this innovative computational method, which has potential diagnostic value in cognitive decline. More importantly, the model provided a ranking of predictors of cognitive impairment which is invaluable for identifying the risk various factors (simple and easily available variables) of cognitive impairment in daily life, for effective prevention and intervention to promote healthy aging [23].

## Methods

### Dataset and Participants

The data for this study were obtained from the CHARLS from 2011 to 2015. This longitudinal survey covered 450 villages or communities in 150 counties/districts, of which 52.67% comprised rural areas, and 47.33% was urban areas [33]. The CHARLS survey aims to build high-quality public databases of individuals and families of middle-aged and older persons aged 45 and above across the country. The national baseline survey was conducted in 2011, whereas the second and third data surveys were carried out in 2013and 2015, respectively. A total of 19,817 respondents were involved in the 2011 baseline survey. In 2013, a total of 18,605 respondents participated in the assessment of cognitive ability. In 2015, a total of 21,095 respondents participated in all the same surveys as the baseline survey. First, the 2011, 2013, and 2015 data were merged according to the principle of ID and household ID matching, and a total of 4,043 participants participated in the survey from 2011 to 2015. Then, missed follow-up data and interviewees answered by others, and variables missing >20% of patient data were excluded from the analysis. Finally, respondents who participated in all three surveys and without the characteristics in the aforementioned exclusion criteria were included in the analysis. The final sample size was 2,326. (The data preprocessing process is shown in Supplementary Figure S1). Specifically, basic information module, health status and functioning module, physical examination and blood-based biomarkers data from 2011 to 2015, and cognitive module data from 2013 were used in this study. The study protocol of the CHARLS was approved by the Peking University Biomedical Ethics Committee, which conformed to the standards set by the latest revision of the Declaration of Helsinki (IRB00001052-11015) (http://charls.ccer.edu.cn/charls/, https://opendata.pku.edu.cn/dataverse/CHARLS).

### Patient and Public Involvement

In this study, we used data from open database CHARLS, which is a nationally longitudinal survey. Therefore, no direct patient was involved and contacted.

### Cognitive Function

To measure the cognitive status of the research population, several measurements of the telephone interview of cognitive status (TICS-10) in the CHARLS data were used [34, 35]. These included date, week, and season among others (orientation and attention), which scored with 5 points; 100 minus 7 calculation series scored with 5 points; the recall, delayed word recall and episodic memory for 10 words was scored with 20 points; and the drawing of two repeated five-sided graphs (visual spatial abilities) was scored with 1 point. The total cognitive function score was 31 points. In general, a higher score indicated better cognitive function of middle-aged and elderly people.

Based on the previous studies, the results of cognitive classification and baseline demographics (e.g., age, education level, marital status, type of residence), health status and functioning (ADL, IADL, functional limitations, life expectancy, eyes, hearing, oral cavity, pain, physical examination, and blood indicators), emotional status (depression CES-D), lifestyle and behavior (sleeping, physical activity, social interaction, smoking, and drinking) were related. Therefore, 44 baseline features in the model were selected to reflect the previously determined predictors in the dataset.

### Demographics

Demographic variables included gender, age, education (no formal education illiterate, does not finish primary school but capable of reading or writing, sishu, elementary school, middle school, vocational school, two/three-year college/associate degree, four-year college/bachelor’s degree, Post-graduate, Master’s degree), household registration (agricultural household registration, non-agricultural household registration and unified residence household registration), marital status (being married and not being married) among others. Six options were present in the questionnaire regarding marital status, namely “married and living with spouse,” “married but not living with a spouse for a temporary period due to work and other reasons,” “separated,” “divorced,” “widowed,” and “never married.” The first two types of marital status were defined as “being married,” while the remaining two types were defined as “not being married.”

### Health Status and Functioning

In this section, to assess health status and functioning, predictive variables mainly related to health and function were used, including eyesight (close) and eyesight (distant objects), hearing problem, tooth loss, chronic disease (participants self-reported whether they had a chronic disease diagnosed by a doctor), pain (are you often troubled with body pains?), physical functions (height, weight, BMI, and respiratory function, blood pressure), blood indicators, activities of daily living (ADL) and instrumental activities of daily living (IADL) [36]. ADL includes dressing, bathing, eating, getting into and out of bed, using the toilet, controlling urination and defecation and IADL includes doing household chores, preparing hot meals, shopping, money management, taking medicine). Are there any difficulties in these daily routines? There are 4 options for all questions: 1. No difficulty, 2. Difficulty but achievable, 3. Difficulty and need help, 4. Unable to complete. A higher score indicates lower quality of acting.

Functional limitations, included “running 1 km,” “walking 100 m,” “sedentary standing up,” “climbing stairs,” and “picking up coins” and nine questions regarding the difficulty of nine basic activities, with a total score range within 0–27 points. A higher score indicated deteriorating body function Following that life expectancy was assessed through the possibility of an individual’s assumption about living until the expected age. Accordingly, the questions comprised a rating of 1–5, which indicated from “nearly impossible” to “very certain.”

Venous blood samples (biomarkers) comprised: high-sensitivity C-reactive protein (hsCRP), glycosylated hemoglobin (HbA1c), total cholesterol, high density lipoprotein (HDL) cholesterol, low-density lipoprotein (LDL) cholesterol, triglycerides, glucose, blood urea nitrogen (BUN), creatinine, uric acid, and cystatin C [37, 38].

### Emotional Status

The CHARLS database employed the Center for Epidemiologic Studies Depression Scale-10 (CESD-10) to investigate the depression risk among middle-aged and elderly people, with depression scores ranging from 0 to 30 points. Subsequently, higher score indicated higher susceptibility to depression. A score of 10 points or higher indicates high risk of depression [39].

### Lifestyle and Behavior

This study identified lifestyle and behavior as the main factors affecting cognitive function. The other factors identified included sleeping habits (night sleep time and nap time), eating habits (number of meals a day), smoking, drinking, physical activity (amount of exercise per person), energy expenditure (total number of high and low activities of each person * weight), and social interaction (entertainment activities, service activities, other activities, and whether to participate in social activities).

### Outcome Variables

During the 2013 and 2015 follow-ups, the outcome variables of the survey were consistent with the cognitive function test in 2011 variables such as date, week, and season (orientation and attention), and scored 5 points; 100 minus 7 calculation series, scored 5 points, recall and delayed recall word recall episodic memory 10 words, scored 20 points, and drawing two repeated five-sided graphs (visual spatial abilities) scored 1 point. The total cognitive score was 31 points. When the total score of participants exceeded 1 standard deviation lower than the standard of the corresponding age group, the participants were classified as cognitively impaired. Meanwhile, other participants were defined as having normal cognition [34, 40].

### Performance Evaluation and Data Analyses

All data were analyzed using SPSS version 26.0 version and Python version 3.8. The random forest and logistic regression model were employed to predict the cognitive function of middle-aged and elderly people in 2013 and 2015. Subsequently, 2,326 data sets were divided into a training set (70%, N = 1,628) and test set (30%, N = 698). Some missing values were imputed using the method of nearest neighbor imputation. A flow chart of the random forest data analysis process is shown in Supplementary Figure S1. The parameters set of the random model were as follows: maximum depth of the forest = 6, and maximum number of leaves = 90(to alleviate over-fitting). The learning rate was set to 0.001, and the training evaluation index was 100 iterations of AUC training. When the number of iterations exceeded 5 times, no further increase in the AUC value was observed, and the training was stopped to prevent over-fitting. To validate the model, the 10-fold cross-validation method was used.

In the logistic regression model, the input data were standardized in order to speed up the gradient descent to find the optimal solution. The regularization parameter was selected as “L2,” the number of cross-validation was set to 10 folds, the loss function was optimized by the second derivative matrix of the loss function, the regularization coefficient was set to 20 equal parts from −2 to 2, and the error range of the iteration termination criterion was 0.01.

#### Descriptive Analysis

Results were expressed as the mean (± standard deviation) of continuous variables or the percentage of subjects in categorical variables. The AUC value in the Receiver Operator Characteristic curve (ROC) was the area under the ROC curve, which reflected the performance of the model. The AUC >0.9 was considered very good, 0.8–0.90 was considered good, 0.7–0.8 was regarded as fair, and <0.7 was regarded as poor [41].

## Results

### Sample Characteristics

The baseline characteristics of this study in 2011 are presented in Table 1. In the first year (baseline, 2011), there were 2,326 participants, including 1,318 people aged 45–59 (middle-aged) and 1,008 people aged 60 and above (elderly people). The average score of cognitive function was 12.94 (±5.95). The specific score for each baseline or variable was as follows: demographic (5), health status (24), functioning (3), emotional status (2), and lifestyle and behavior (10).

**TABLE 1:** Baseline sample characteristics (China, 2011).

Variable	M(SD) or N(%)
Age	58.64 (8.69)
45–59 years old(N = 1,318)	52.37(4.41)
60+ years old(N = 1,008)	66.83(5.54)
Gender	
Male	1,083 (46.60%)
Female	1,243 (53.4%)
Education	
No formal education illiterate	664 (28.50%)
Did not finish primary school but capable of reading or writing	532 (22.90%)
Elementary school	569 (24.50%)
Middle school	381 (16.40%)
High school and above	180 (7.70%)
Hukou	
Agricultural	2011 (86.50%)
Non-agricultural	307 (13.20%)
Unified residence	8 (0.30%)
Marital status	
Married	2083 (89.60%)
Not married	243 (10.40%)
Health status and functioning	
Physical examination	
BMI	23.23 (3.88)
Central obesity	
Yes	520 (22.40%)
No	1806 (77.60%)
Breath (vital capacity)	257.24 (109.81)
Systolic	128.97 (21.32)
Diastolic	75.40 (12.33)
Pulse	72.01 (10.26)
Vision	
Eyesight (close)	3.85 (0.87)
Eyesight (distance)	3.80 (0.94)
Hearing	3.62 (0.89)
Tooth loss	
Yes	188 (8.10%)
No	2,138 (91.90%)
Chronic diseases	
Yes	1,633 (70.20%)
No	693 (29.80%)
Pain	
Yes	854 (36.70)
No	1,472 (63.30%)
Biomarkers	
HsCRP	2.59 (6.58)
HbA1c	5.25 (0.73)
Total cholesterol	192.47 (37.43)
HDL cholesterol	52.12 (15.10)
LDL cholesterol	114.36 (34.16)
Triglycerides	125.54 (90.27)
Glucose	106.90 (32.12)
Blood urea nitrogen (BUN)	15.85 (4.73)
Uric acid	4.51 (1.22)
Cystatin C	1.02 (0.24)
Creatinine	0.78 (0.18)
Life expectancy	2.96 (1.03)
Functioning	
Functional limitations	10.90 (4.13)
ADL	4.57 (3.20)
IADL	5.59 (1.72)
Emotional status	
Depression (CESD-10)	8.86 (6.42)
High depression risk	
Yes	821 (35.30%)
No	1,505 (64.70%)
Lifestyle and behavior	
Night sleep (Hour)	6.28 (1.938)
Nap time (Minute)	31.27 (44.50)
Eating habits	3.14 (0.40)
Smoking	
Yes	876 (37.70%)
No	1,450 (62.30%)
Drinking	
Yes	764 (32.80%)
No	1,562 (67.20%)
Physical activity	2,589.33 (4578.68)
Energy consumption	145495.67 (260370.71)
Social interaction	
Entertainment	0.55 (0.72)
Service activities	0.10 (0.33)
Other activities	0.02 (0.5)
Cognitive function	
2011Cognitive total score	12.94 (5.95)

### Model Performance

Table 2 shows the comparison of AUC between random forest and logistic regression. The results show that random forest performs better than logistic regression. Table 3 illustrates the model performance indicators of random forest models. The results for the 2nd and 4th year follow-up model indicators show that the AUC of the 3 ML models (model a^1^, model a^2^, model b) were 0.81, 0.79, and 0.80, respectively (see Supplementary Figures S2–S4). The models fit was good and fair.

**TABLE 2:** AUC of random forest and logistic regression (China, 2022).

		Mean AUC	95%CI	Model fit
Random models	Model a^1^	0.81	0.79–0.83	Good
	Model a^2^	0.79	0.76–0.82	Fair
	Model b	0.80	0.77–0.83	Good
Logistic regression	Model a^1^	0.61	0.58–0.64	Poor
	Model a^2^	0.62	0.59–0.65	Poor
	Model b	0.70	0.67–0.73	Fair

**TABLE 3:** Model performance (China, 2022).

	Model a^1^	Model a^2^	Model b
Precision	0.69	0.62	0.84
Recall	0.83	0.79	0.79
F-score	0.75	0.69	0.72
Accuracy	0.82	0.79	0.84

Note: 1) Model a^1^refers to the model indicators of the predictive model constructed by the independent variables in 2011 to predict the cognitive impairment in 2013.

2) Model a^2^ refers to the model indicators of the predictive model constructed by the independent variables in 2011 to predict the cognitive impairment in 2015.

3) Model b refers to the model indicators of the prediction model constructed by the cross-sectional data in 2015 and the independent variables to predict the cognitive impairment in 2015.

### Predictor Variables Importance

Table 4 presents the predictor variable ranking results by importance of 10 feature selection methods that were performed on the dataset. The cognitive classification prediction model for 2013 ranked the variables from the most important to the lest important as follows: “education,” “triglycerides,” “age,” “BMI,” “LDL cholesterol,” “uric acid,” “functional limitations,” “pulse,” “HDL cholesterol,” and “life expectancy.” For 2015, the prediction of cognitive classification model’s variable ranking was in the following order: “BMI,” “depression,” “pulse,” “systolic,” “education,” “total cholesterol,” “blood urea nitrogen (BUN),” “HDL,” “cholesterol,” “Uric acid,” and “HsCRP.” The important factors of the forecast model for the verification of horizontal data were organized in the following order: “BMI,” “pulse,” “depression,” “breath,” “creatinine,” “age,” “night sleep time,” “education,” “triglycerides,” and “total cholesterol.”

**TABLE 4:** Importance of variables of predictive models and validated predictive models (China,2022).

	Model a^1^	Importance	Model a^2^		Model b	Importance
1	Education	0.0702	BMI	0.0809	BMI	0.0883
2	Triglycerides	0.0694	Depression	0.0583	Pulse	0.0523
3	Age	0.0673	Pulse	0.0508	Depression	0.0521
4	BMI	0.0651	Systolic	0.0503	Breath	0.0481
5	LDL cholesterol	0.0563	Education	0.0462	Creatinine	0.0462
6	Uric acid	0.0561	Total cholesterol	0.0439	Age	0.0407
7	Functional limitations	0.0525	Blood urea nitrogen (BUN)	0.0405	Night time	0.0399
8	Pulse	0.0454	HDL cholesterol	0.0398	Education	0.0396
9	HDL cholesterol	0.0444	Uric acid	0.0384	Triglycerides	0.0392
10	Life expectancy	0.0407	HsCRP	0.0362	Total cholesterol	0.0382

Note: Model a^1^, Model a^2^ and model b have the same meaning as the above table.

The importance of variables in this study is based on the contribution of features in each decision tree. The average contribution of all decision trees is the importance of the feature. The importance of the features in the decision tree depends on the change of the Gini coefficient of the nodes of the decision tree. The top ten features were screened and their relative importance was re-stated.

## Discussion

### Advantages of Machine Learning Methods

With the advances in the electronic age, the application of machine learning to clinical disease diagnosis, differential diagnosis, and disease prediction is increasing [42, 43]. This study provides a new method of machine learning to predict cognitive impairment in middle-aged and elderly people. The method of mutual verification of the combination of longitudinal and cross-sectional data was adopted to improve the effectiveness of the model. Good results were achieved, with the best model producing an AUC of 0.81 [41] compared with logistic regression [22, 42].

Indeed, logistic regression was previously considered a standard method for binary classification. Compared with machine learning methods, logistic regression is limited by assumptions of normality and linear relationships and may not evaluate the non-linear and complex relationships between physiological and social data. As a non-parametric technique, random forest in machine learning can overcome the shortcomings of under-fitting in traditional regression methods, and at the same time prevent over-fitting [11, 44]. It is thus considered a more flexible method for assessing complex interactions between variables. This study also corroborates previous findings that random forest is more suitable for the application of high-dimensional variables and large-scale data [45]. At present, it has become an alternative standard classification method to logistic regression [12]. Couronné et al. used 243 real data sets to conduct systematic large-scale comparative study, which showed that the average prediction performance of random forest is better than that of logistic regression [46]. In view of the fact that there are many variable dimensions involved in this study, the sample size was large. Predictive models for cognitive impairment in middle-aged and elderly people are lacking. Therefore, the present study established a predictive model of cognitive impairment in middle-aged and elderly people using the random forest method. The results indicated that the use of sociodemographic characteristics, health status and functioning, and emotional state could accurately predict cognitive impairment.

### Impact of Demographic Variables on Cognitive Impairment

From the results, the demographic variables of age and education level are important predictors, which is similar to previous research results [22, 47]. Previous studies showed that the prevalence of dementia was higher among the people aged 65 years old or older [47]. Education level could reduce the risk of cognitive impairment and dementia [48, 49]. Compared with people with no education, less education was associated with a lower risk of cognitive impairment. Notably, education contributes to cognitive reserve [50, 51].

### Impact of Health Status on Cognitive Impairment

Blood predictors including non-invasive markers were used in the current research to predict cognitive impairment. Non-invasive markers help predict patients with normal cognitive status or cognitive impairment, which may also contribute to better preventive measures [52, 53]. The results of this study indicated that Biomarkers in health status are important risk factors for predicting cognitive impairment. This finding is consistent with previous studies. We also found that uric acid, HsCRP, creatinine, LDL cholesterol, HDL cholesterol, total cholesterol, triglycerides, and BUN in venous blood samples were important predictors of the cognitive impairment. This finding is consistent with previous studies showing that low uric acid is a risk factor for cognitive impairment. When at an appropriate level, uric acid could reduce the occurrence and development of cognitive impairment [54]. Decreased creatinine concentration may indicate the occurrence of cognitive decline [55].

The results of epidemiological studies indicated the presence of a preliminary correlation between inflammation and cognitive impairment [56]. Noble, Manly [57] reported that the elderly with higher C-reactive protein levels are at a higher risk of memory impairment. Therefore, evidence supports the important role of this biomarker as a vascular risk factor for cognitive decline [58]. Furthermore, a 31-year longitudinal study concluded that the HsCRP changes during the middle age could reflect the underlying process of aging-related cognitive decline [59]. Longitudinal studies also found that higher baseline Triglycerides and LDL-C concentrations were associated with a higher rate of cognitive decline, but the effect of Triglycerides was not significant [60, 61]. Overall, our findings were slightly different from those of previous studies. Notably, HDL cholesterol and triglycerides showed an important predictive effect on cognitive impairment. The latest meta-analysis experiments have concluded that triglycerides potentially affect cognition [62–64].

For the indicators of physical examination, the findings of this study were in line with the previous findings that physiology is correlated with cognitive function was consistent. A useful indicator of physical health status is BMI, which screens for human weight. Furthermore, BMI ranked higher in terms of the important features for both regardless of it being the longitudinal prediction model and horizontal verification model. A longitudinal study conducted in South Korea found that obesity or weight loss in the later stages of life did not affect the risk of cognitive impairment [65]. Another study found that obesity at the middle age was an important predictor of the development of cognitive impairment in the later life phase [66]. In the latest research, it was proposed that being underweight was possibly an important risk factor for cognitive impairment among the elderly in China [26]. The results of a machine learning study also show that BMI ranked among the top 10 predictors of mild cognitive impairment (MCI) [21, 67]. Therefore, interventions for cognitive function among the elderly should target weight management.

The importance of blood pressure was identified. Similarly, previous studies highlighted a positive association between elevated diastolic or systolic blood pressure and the risk of cognitive impairment [68]. This condition played a crucial role in the guidance of routine clinical practice. To illustrate, effective control of blood pressure could reduce the risk of cognitive impairment, which was in line with previous research results [69]. Furthermore, in the present study systolic pressure was an important predictor of cognitive impairment, and has been established to cause cerebrovascular diseases and subsequently reduce cognitive ability [70, 71]. Pulse is also an important predictor. Previous studies also that showed the combination of higher pulse speed and age contributed to a gradual decrease in cognitive ability [72].

In a meta-analysis, a negative correlation was found between pulse wave velocity and cognition, particularly executive function, memory, and overall cognition. However, this association was independent of demographic, clinical, and evaluation characteristics [73]. In all horizontally verified prediction models, the feature of the breath (vital capacity) function was ranked fourth. Previous studies have also verified the relationship between lung function and cognitive function. A recent systematic review reported that although some research has shown a correlation between lung function and cognition, the result of the current study indicated several limitations and used a single measurement method [74]. Similarly, the present study, used only one type of expiratory volume as an indicator of lung function. Thus, further study is required to unravel the complex relationship among these factors.

Functional limitations have been proved as important predictors of cognitive impairment in previous studies. Multiple horizontal and longitudinal studies have concluded that poorer dysfunction is associated with more severe cognitive impairment [75, 76]. In recent years, functional limitations have been added to the cognitive impairment screening test [77, 78]. This proves that the negative effect of functional limitation in cognitive dysfunction is adequate.

### Effect of Depression on Cognitive Impairment

The present study demonstrated that depression is an important predictor of cognitive impairment. In the predictive model, depression was ranked first in importance. Furthermore, the negative effect harm of depression has been proven in multiple studies, including accelerating individual cognitive impairment [29–31, 79, 80]. Depressive symptoms reflect the individual’s emotional state, and the 2-year predictive model demonstrated that life expectancy is also an important factor in the predictive model. Our results showed that life expectancy is one of the important factors affecting cognitive impairment. In fact, life expectancy refers to the possibility for middle-aged and elderly people to envision living until the expected age is reached, a higher life expectancy reflects a positive emotional state. Previous research found that positive emotions are more likely to protect against cognitive decline, whereas negative emotions are associated with a higher risk of mild cognitive impairment and dementia [81]. Therefore, relevant literature has suggested that dementia interventions based on established positive psychology principles could help elderly people cope with their diseases [82, 83]. The concept of improving cognitive function through psychosocial interventions is now also gaining acceptance [84].

### Influence of Lifestyle and Behavior on Cognitive Function

In this study, night time sleep, as in lifestyle and behavior factor, played an important role in the horizontal data prediction model. Previous studies demonstrated that poor sleep quality was associated with poor cognitive function [85, 86]. As a solution, behavioral intervention to improve sleep could effectively improve the cognitive ability of patients with cognitive impairment [86], proving that night time sleep is important for the cognitive function of middle-aged and elderly people.

### Conclusion

In conclusion, this study has proved the accuracy of ML using vertical and cross-sectional data. Importantly, this study added the measure of non-invasive marker blood. This survey has proved the utility of ML in long-term predictions of persistent cognitive impairment and identifications risk markers, supporting testing of new ML algorithms to predict disease progression. Furthermore, the availability of these variables suggests their potential use in screening for future cognitive impairment among the elderly in the community. The risk factors discovered could also be used by clinical staff to treat cognitive impairment and develop intervention programs. More importantly, the predictors of cognitive impairment found in this study through predictive models can be subjects of public education to reduce their effect on cognitive impairment in daily life. This is of great significance for preventing the deterioration of the quality of life of middle-aged and elderly people and promoting the healthy aging of society.

### Limitation

There are some limitations in this study. First, this study only used the method of random forest in comparison with logistic regression. There is a lack of comparison with other methods, such as the support vector machine method. Although the advantages of random forest have been demonstrated in other studies, further comparison with other methods is needed. Second, although our model screened the important risk factors for cognitive impairment, the specific relationship between these risk factors and cognitive impairment, whether being positive or negative, remained unclear. Therefore, further research is required to elucidate the value of these variables in cognitive impairment. Third, the measure of cognitive impairment in this study was self-reported by division respondents rather than derived from clinical judgment. While this is a good way for the community to implement measurements, there is a risk of pseudocognitive impairment. Therefore, future research should incorporate clinical reporting to refine the findings.

## Supplementary Material

Supplementary Figure S1 Flowchart of data preprocessing and random forest data analysis experiment (China, 2022).

Supplementary Figure S2 Receiver operating characteristic curve of 2-year (2013). The area under the receiver operating characteristics curve (AUC) is 0.81 (China, 2022).

Supplementary Figure S3 Receiver operating characteristic curve of 4-year (2015). The area under the receiver operating characteristics curve (AUC) is 0.79 (China, 2022).

Supplementary Figure S4 Receiver operating characteristic curve of cross-sectional verification data (2015). The area under the receiver operating characteristics curve (AUC) is 0.80 (China, 2022).

## Abbreviations

ADL, activities of daily living; CHARLS, China health and retirement longitudinal study; IADL, instrumental activities of daily living; ML, machine learning; TICS, telephone interview of cognitive status.
