# Corrigendum: Using Machine Learning to Predict Cognitive Impairment Among Middle-Aged and Older Chinese: A Longitudinal Study

**DOI:** 10.3389/ijph.2023.1606127

**Published:** 2023-05-18

**Authors:** Haihong Liu, Xiaolei Zhang, Haining Liu, Sheau Tsuey Chong

**Affiliations:** ^1^ Centre for Research in Psychology and Human Well-being, Faculty of Social Sciences and Humanities, Universiti Kebangsaan Malaysia, Bangi, Malaysia; ^2^ Department of Psychology, Chengde Medical University, Chengde, China; ^3^ Department of Biomedical Engineering, Chengde Medical University, Chengde, China; ^4^ Faculty of Engineering, Universiti Putra Malaysia, Serdang, Malaysia; ^5^ Hebei Key Laboratory of Nerve Injury and Repair, Chengde Medical University, Chengde, China; ^6^ Hebei International Research Center of Medical Engineering, Chengde Medical University, Chengde, China; ^7^ Counselling Psychology Programme, Secretariat of Postgraduate Studies, Faculty of Social Sciences and Humanities, Universiti Kebangsaan Malaysia, Bangi, Malaysia

**Keywords:** longitudinal study, machine learning, random forest, middle-aged and older Chinese, cognitive impairment, dementia

There was an error in the **Funding** statement. The authors erroneously included the funders “Humanities and Social Science Research Project of Hebei Education Department (SQ2022059), and University-Level Scientific Research Project in CDMC (202113).”

The authors apologize for this error and state that this does not change the scientific conclusions of the article in any way. The first published version of the article has been updated.

