# Supplementary figures and images for: Using Machine Learning to Predict Cognitive Impairment Among Middle-Aged and Older Chinese: A Longitudinal Study
Source: Int J Public Health. 2023 Jan 19;68:1605322. doi: 10.3389/ijph.2023.1605322 (PMC9926933; doi:10.3389/ijph.2023.1605322)

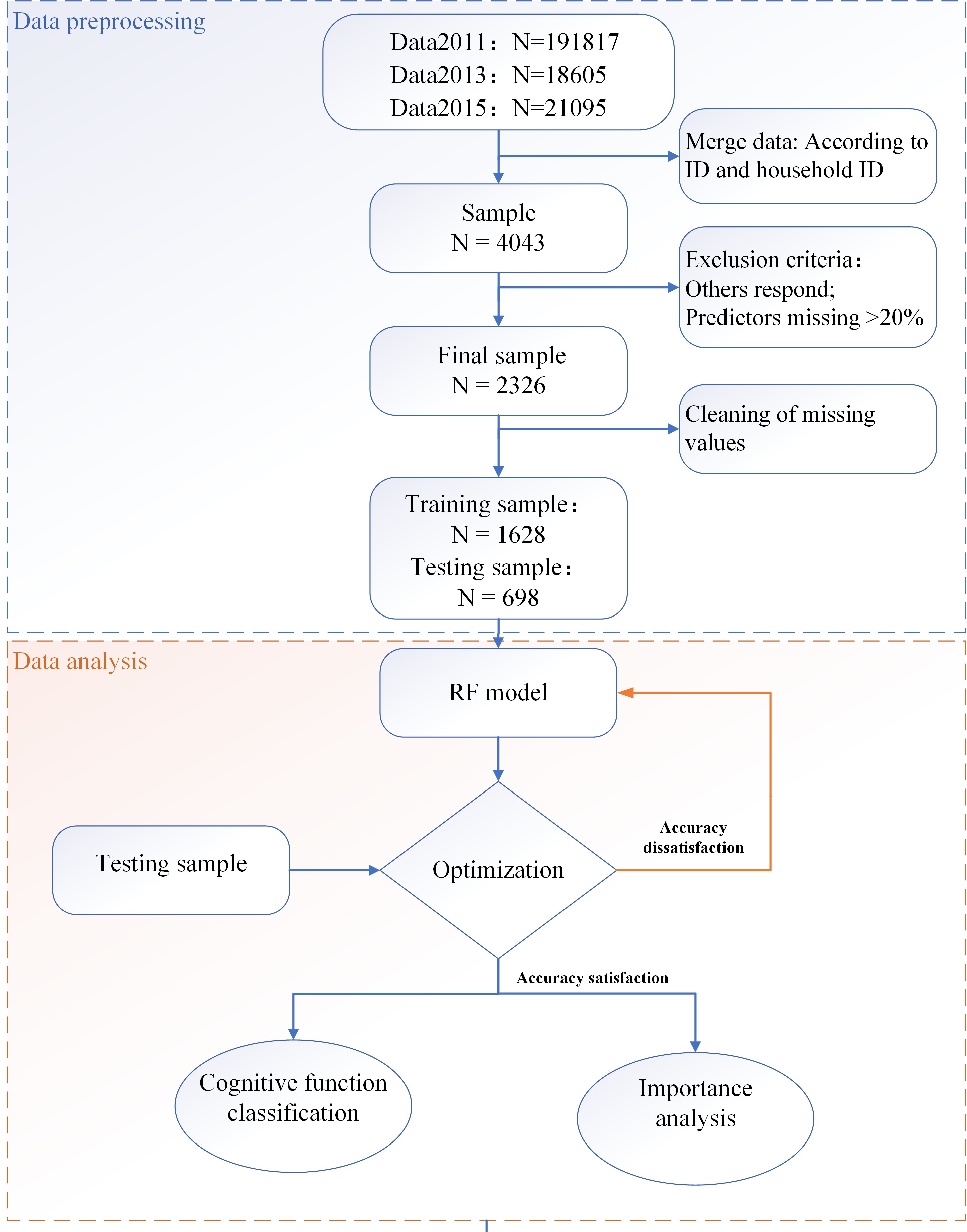

Supplement: Supplementary file 4 [file Image1.jpg]
